# Supplementary material for: The molecular structure of IFT-A and IFT-B in anterograde intraflagellar transport trains
Source: Nat Struct Mol Biol. 2023 Jan 2;30(5):584–93. doi: 10.1038/s41594-022-00905-5 (PMC10191852; doi:10.1038/s41594-022-00905-5)
Supplement: Supplementary file 1 — Reporting Summary [file 41594_2022_905_MOESM1_ESM.pdf]

nature portfolio

Corresponding author(s): Gaia Pigino

Last updated by author(s): 2022/11/30

Reporting Summary

Nature Portfolio wishes to improve the reproducibility of the work that we publish. This form provides structure for consistency and transparency in reporting. For further information on Nature Portfolio policies, see our Editorial Policies and the Editorial Policy Checklist.

Statistics

For all statistical analyses, confirm that the following items are present in the figure legend, table legend, main text, or Methods section.

n/a	Confirmed	
☒	☐	The exact sample size (*n*) for each experimental group/condition, given as a discrete number and unit of measurement
☒	☐	A statement on whether measurements were taken from distinct samples or whether the same sample was measured repeatedly
☒	☐	The statistical test(s) used AND whether they are one- or two-sided<br>*Only common tests should be described solely by name; describe more complex techniques in the Methods section.*
☒	☐	A description of all covariates tested
☒	☐	A description of any assumptions or corrections, such as tests of normality and adjustment for multiple comparisons
☒	☐	A full description of the statistical parameters including central tendency (e.g. means) or other basic estimates (e.g. regression coefficient) AND variation (e.g. standard deviation) or associated estimates of uncertainty (e.g. confidence intervals)
☒	☐	For null hypothesis testing, the test statistic (e.g. *F*, *t*, *r*) with confidence intervals, effect sizes, degrees of freedom and *P* value noted<br>*Give P values as exact values whenever suitable.*
☒	☐	For Bayesian analysis, information on the choice of priors and Markov chain Monte Carlo settings
☒	☐	For hierarchical and complex designs, identification of the appropriate level for tests and full reporting of outcomes
☒	☐	Estimates of effect sizes (e.g. Cohen's *d*, Pearson's *r*), indicating how they were calculated

Our web collection on statistics for biologists contains articles on many of the points above.

Software and code

Policy information about availability of computer code

Data collection	Cryo-electron tomograms were collected with Serial-EM V4.0
Data analysis	Subtomogram averaging was performed with STOPGAP V0.7.1, Warp/M V1.0.9 and Relion V3.1.3. Structural prediction performed with Alphafold V2.1.1. MDFF performed on the NAMDinator web server (https://namdinator.au.dk/).

For manuscripts utilizing custom algorithms or software that are central to the research but not yet described in published literature, software must be made available to editors and reviewers. We strongly encourage code deposition in a community repository (e.g. GitHub). See the Nature Portfolio guidelines for submitting code & software for further information.

Data

Policy information about availability of data

All manuscripts must include a data availability statement. This statement should provide the following information, where applicable:

- Accession codes, unique identifiers, or web links for publicly available datasets
- A description of any restrictions on data availability
- For clinical datasets or third party data, please ensure that the statement adheres to our policy

The following maps have been deposited to the Electron microscopy data bank: IFT-B consensus of focused refinements (EMD-15977), IFT-B1 focused refinement (EMD-15978, IFT-B1 peripheral focused refinement as associated map), IFTB2 focused refinement (EMD-15979, IFT-B2 peripheral focused refinement as associated

map), IFT-B low-resolution overall map to validate consensus (EMD-16014) and IFTA (3-repeat map EMD-15980, 1 repeat and masked refinements as associated maps in this deposition). The IFT-B and IFT-A atomic models have been deposited to the protein data bank with the codes PDB-8BD7 and PDB-8BDA respectively.

Human research participants

Policy information about studies involving human research participants and Sex and Gender in Research.

Reporting on sex and gender	N/A
Population characteristics	N/A
Recruitment	N/A
Ethics oversight	N/A

Note that full information on the approval of the study protocol must also be provided in the manuscript.

Field-specific reporting

Please select the one below that is the best fit for your research. If you are not sure, read the appropriate sections before making your selection.

☒ Life sciences ☐ Behavioural & social sciences ☐ Ecological, evolutionary & environmental sciences

For a reference copy of the document with all sections, see nature.com/documents/nr-reporting-summary-flat.pdf

Life sciences study design

All studies must disclose on these points even when the disclosure is negative.

Sample size	We kept acquiring tomograms and adding subtomograms to our average until the overall resolution, as assessed by the gold-standard Fourier Shell Correlation (FSC) 0.143 criteria, stopped improving with the addition of more data.
Data exclusions	3D Classification was performed in Relion to remove subtomograms that did not contribute to the overall average.
Replication	Cryo-EM grids used for data collection were frozen on 7 separate occasions (7 separate cultures = 7 biological replicates).
Randomization	Gold standard refinement includes randomisation into two groups and independent refinement in Relion.
Blinding	N/A because no variables/conditions were investigated in our study.

Reporting for specific materials, systems and methods

We require information from authors about some types of materials, experimental systems and methods used in many studies. Here, indicate whether each material, system or method listed is relevant to your study. If you are not sure if a list item applies to your research, read the appropriate section before selecting a response.

Materials & experimental systems

n/a	Involved in the study	
☒	☐	Antibodies
☐	☒	Eukaryotic cell lines
☒	☐	Palaeontology and archaeology
☒	☐	Animals and other organisms
☒	☐	Clinical data
☒	☐	Dual use research of concern

Methods

n/a	Involved in the study	
☒	☐	ChIP-seq
☒	☐	Flow cytometry
☒	☐	MRI-based neuroimaging

Eukaryotic cell lines

Policy information about cell lines and Sex and Gender in Research

Cell line source(s)	Chlamydomonas reinhardtii wild-type (CC625) cells and CC625 cells with glycocalyx proteins FMG1A and FMG1B deleted by CRISPR

Authentication	PCR validation of cell lines.
Mycoplasma contamination	N/A
Commonly misidentified lines<br>(See ICLAC register)	N/A
